# Mood Regulation Focused CBT Based on Memory Reconsolidation, Reduced Suicidal Ideation and Depression in Youth in a Randomised Controlled Study

**DOI:** 10.3390/ijerph15050921

**Published:** 2018-05-05

**Authors:** Göran Högberg, Tore Hällström

**Affiliations:** 1 Child Psychiatry, BUP Globen, Stockholm 12177, Sweden; 2 Department of Psychiatry and Neurochemistry, Institute of Neuroscience and Physiology, The Sahlgrenska Academy at the University of Gothenburg, 43141 Mölndal, Sweden; tore.hallstrom@gu.se

**Keywords:** suicide, secondary prevention, adolescents, therapy, counter-conditioning, memory reconsolidation

## Abstract

Suicide attempts and suicidal ideation in adolescence are considered to be related to suicide and psychiatric adversity later in life. Secondary prevention by improving the treatment of suicidal youth is a distinct possibility. In this study, treatment with a systematised mood-regulation focused cognitive behavioural therapy (MR-CBT) (n = 15) was compared with treatment as usual (TAU) (n = 12) in a group of depressed adolescents in a clinical setting. MR-CBT focuses on mood regulation by means of counter conditioning with memory reconsolidation being the proposed mechanism of change. Subjects practice keeping emotionally positive memories to diminish the emotional impact of negative memories. Symptoms of depression were tested with a short version of the Mood and Feelings Questionnaire (SMFQ), and wellbeing with the World Health Organization 5 Wellbeing Index (WHO-5). Suicidal events were rated according to the clinical interview Columbia Suicide Severity Rating Scale (C-SSRS). Suicidal events at the end of treatment were significantly reduced in the MR-CBT group, but not in the TAU group. Depression and wellbeing improved significantly in both treatment groups. While far from conclusive, the results are encouraging enough to suggest that further studies should be undertaken to examine whether psychotherapy focusing on mood regulation for young individuals at risk might enhance secondary prevention of suicide.

## 1. Introduction

Globally, suicide is the second cause of death in the 15–29 years age group [1]. It has been shown that risk for suicide in young adulthood often had a beginning in adolescence [2]. Suicide attempts in adolescence are also associated with worse psychiatric, medical and social outcomes in adulthood [3].

In order to achieve secondary prevention of both suicide and psychiatric adversity there is a need for efficacious treatment of depressed and suicidal youth [4]. In a meta-analysis of psychotherapy with depressed youth it was noted that the mean effect was 0.29, which is low, and that there was no difference in effect between cognitive and non-cognitive treatments [5]. This provides the rationale for efforts to develop more efficient psychological treatments for depressed youth. In order to identify more robust treatments, Weisz [6] suggested psychotherapy research focused on actual clinical care. Using suicide attempts and non-suicidal self-harm as follow up measures, Ougrin et al. [7] found that there was no statistically significant difference between specific psychological interventions and standard care. However, Asarnow et al. [8] found that a safety promoting approach with elements of CBT and family components proved to be advantageous regarding suicide prevention among high-risk youths.

Depressive symptoms can occur in connection with both psychological and somatic problems. For instance, mononucleosis may result in a depressive state in adolescents. However, with youth it has been concluded that depression for the most part is situational, relating to present and past life experiences [9,10,11,12,13]. An indication of the importance of situational causes of depression and suicidality in adolescents was found in the correlation between PTSD and suicidality in adolescents [14], and increased suicidality was noted in 20% of a persistently unwell depressed group in a 10-year follow-up study [15].

The teenage brain is in a process of maturation and regulatory functions are still not fully developed, as noted by Steinberg [16] (p. 73) this is period of “…suboptimal patterns of development... such as the excessive down-regulation of mood and motivation…or the inadequate control of arousal”. In a comparison with normal subjects it was shown that clinically depressed adolescents experienced both negative and positive affect, but that the problem consisted of difficulty in letting go of negative affect and of maintaining positive affect [17]. There is an increasing focus on the process of emotion regulation in children and adolescents with psychological disorders [18]. Larsen [19] (p. 149) proposed a theory based on control of mood regulation: “We want people to have moods, but we don’t necessarily want moods to have people. That is, people can take control of their mood states, regulate how much and how long particular mood states will persist, and regulate the expression of particular moods”. Among treatment seeking adolescents it was found that difficulties in experiencing positive affect and a high intensity of negative affect was correlated with increased suicidality [20]. Dysfunctional processing of autobiographical memories such as bias for negative emotional memories and difficulties in retrieving positive memories is also found in depressed adults [21]. Hopelessness is associated with suicide attempts and there might be a link between depressed subjects’ selective attention to negative autobiographical memories and the development of hopelessness [22].

Reconsolidation, a term from affective neuroscience, means that an autobiographical memory, when activated, can change its emotional valence in a short time frame and be reconsolidated with new emotional valence as part of personal memory [23]. This means that a recurring negative emotional memory can be changed by applying techniques of safety and control to the retrieval of such a memory, so that it loses some of its negative impact on mood. There is an emerging interest in memory reconsolidation as a mechanism of change in psychotherapy, and several psychotherapy protocols based on memory reconsolidation have been presented [24,25,26,27,28,29]. Treatment focusing on memory reconsolidation offers the prospect of neutralising negative affect stemming from fearful memories. This line of treatment is akin to the principles of Wolpe’s counter-conditioning [30] (p. 71): “If a response antagonistic to anxiety can be made to occur in the presence of anxiety-evoking stimuli so that it is accompanied by a complete or partial suppression of the anxiety responses, the bond between these stimuli and the anxiety responses will be weakened”.

The aim of the present study was to compare a mood regulation focused cognitive behavioural therapy (MR-CBT), a protocol based on memory reconsolidation, with treatment as usual (TAU), with depressed adolescents in a natural child psychiatric setting. The primary outcome variables were suicidality and depression. The secondary outcome variable was wellbeing. The planned number of participants was 52.

## 2. Material and Methods

### 2.1. Setting

The study was conducted at four outpatient units of BUP Child Psychiatric Clinic in Stockholm, from June 2012 to February 2015. Eight therapists from these units were trained during 6 days in the use of the MR-CBT protocol; these were two child psychiatrists, two social workers, and three psychologists. Parents or adolescents contacting the clinic were asked whether they were interested in knowing about the research project and in case they did, were duly informed. Those who wanted to participate were then asked to complete the short version of the Mood and Feelings Questionnaire (SMFQ) [31]. The inclusion criterion was depression according to the SMFQ score, and the exclusion criteria were need of a translator, and refugees lacking a residency permit. There was no blinding of treatment or allocation.

The study was approved by the local ethics committee at the Karolinska Institute, Stockholm, Sweden, (2008/4:9) and informed consent was obtained from participants and their care-takers.

**Figure 1:**
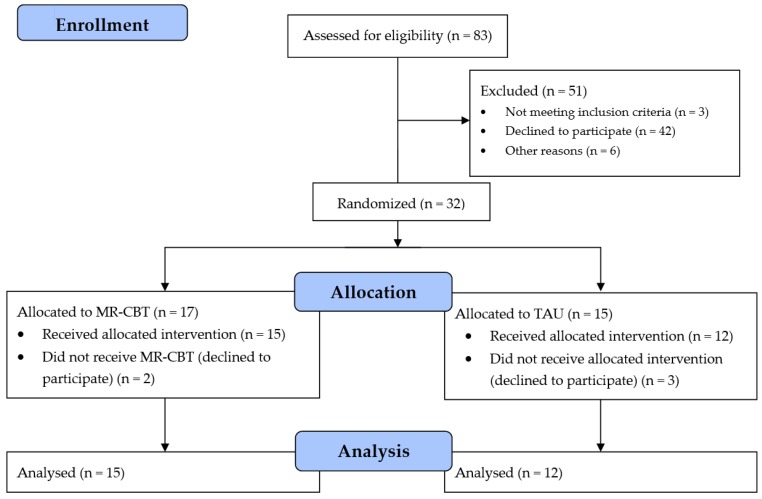
This flowchart shows the flow of participants in the study.

The flow of participants is shown in Figure 1. The participants were a natural clinical sample and treatment was given according to experienced and estimated needs.

### 2.2. MR-CBT

The protocol of the systematised MR-CBT is based on the mechanism of memory reconsolidation, meaning that with evoked activated memories a new affective response can be learned during a short timeframe [23]. The focus in the method is on regulation of moods, with charting a mood map at the start, and on problem solving, with training in keeping positive affect and letting go of negative affect [28]. The mood map was presented in another article [32] as a sketch “…with the person in the centre and the diverse moods encircling. Ways of showing the interactions between the mood states were introduced. The first mood map expressed the present situation, the second the desired future state, and the third what was needed to get there”. The treatment focuses on recent situations, and deals with the memory of those events. There is no search for old events, but when they occur in present moods they are duly taken care of. If there is a problem with sleeping such as nightmares, traumatic flashbacks, grief reactions, future fears, and tiring rumination, these issues are dealt with first. The protocol also allows for a focus on control of the process by the client, with active consent before new steps are taken during the session. Most of the treatment is actually in the hands of the client, employing visual imagery, the content of which does not have to be revealed to the therapist. The basic sequence is step-by-step to establish positive affect, then activate and process the negative affect, and finally to aim for positive future oriented affect. The protocol is also named the plus-minus-plus method, and in this construction, it is similar to counter-conditioning (reciprocal inhibition) [30]. In summary, the proposed aim is to increase the capacity to retain good emotions and to let go of negative emotions by systematically strengthen positive emotions and diminishing negative emotions from autobiographical memories. The protocol can be applied to different technical treatment modalities, for instance talk, art and play therapy, and is also trans-diagnostic, as mood regulation is a core issue in different psychiatric conditions. The treatment was given without any defined frequency but followed clinical needs. The full protocol in English can be found in the Supplementary Materials.

### 2.3. Control Treatment

The control treatment was treatment as usual (TAU). The treatment given was considered good standard practice in child psychiatry.

### 2.4. Randomisation

An assistant at the unit picked an envelope from an even number of sealed envelopes containing either MR-CBT treatment or TAU.

### 2.5. Medication

In both the TAU and MR-CBT treatment groups, medication was given when considered relevant by the client and therapist.

### 2.6. Primary Outcome Measures

Posner et al. introduced the Columbia Suicide Severity Rating Scale (C-SSRS) as a systematic clinical interview, which relies on scales from previous studies to delineate data from clinical interviews and relevant record information. The psychometric properties of C-SSRS have been reported as good and the scale was suggested for use in clinical and research settings [33]. The scale contains subscales on suicidal ideation as well as behaviour. The severity grades of suicidal ideation are: (1) wish to be dead; (2) nonspecific active suicidal thoughts, (3) active suicidal thoughts with methods, (4) suicidal intent, and (5) suicidal intent with a plan. Suicidality in this study was presented as a suicidal event defined as a discrete period of suicidal ideation or a suicide attempt. The rating of C-SSRS was dichotomised in this study into 0 = no suicidal event and 1 = suicidal event based on suicidal ideation grade (3) or higher, and/or a suicide attempt. The C-SSRS was used with data from the suicide evaluation sections of the records in cases of missing observations.

The short 13-item version (SMFQ) of the 33-item Mood and Feelings Questionnaire was developed and tested as a practical short scale for the self-evaluation of depressive symptomatology and is considered to be a reliable instrument for the evaluation of depressive symptoms in children and adolescents [31,34]. The statements are: (1) miserable and unhappy, (2) didn’t enjoy anything, (3) tired, (4) restless, (5) no good, (6) cried a lot, (7) poor concentration, (8) hated myself, (9) bad person, (10) lonely, (11) unloved, (12) never as good as other children; and (13) did everything wrong. The items are rated on a 3-point Likert scale from 0 to 2, with a higher number indicating more symptoms, the maximum score being 26. The cut-off for depression was set at ≥8. Partial remission [35] was set at >50% decrease in the total SMFQ score combined with a final score <8.

### 2.7. Secondary Outcome Measure

The secondary outcome measure was wellbeing assessed with the World Health Organization 5 Wellbeing Index (WHO-5). The WHO-5 is a short scale measuring self-evaluated wellbeing. The five statements are (1) I have felt cheerful and in good spirits, (2) I have felt calm and relaxed, (3) I have felt active and vigorous, I woke up feeling fresh and rested, and (5) My daily life has been filled with things that interest me. The five items are rated on a 6-point Likert scale from 0 to five, five being the most positive. A percentage value is obtained by multiplying the score by 4 and thus arriving at a scale with 100 as best imagined wellbeing and 0 as worst. The internal consistency of the WHO-5 is good and it has been shown to be a valid screening instrument for depressive symptoms in children [36,37].

### 2.8. Statistics

Fisher’s exact test was used to compare the proportion of suicidal events and remission of depressive symptoms. Differences between baseline and endpoint data were estimated using the Wilcoxon matched pairs test, and endpoint score difference between treatments was calculated with the Mann-Whitney test. Significance was set at *p* < 0.05 in a two-tailed test. In the case of a missing value the last value was carried forward according to the principle of intent to treat (ITT). Effect size was calculated as Cohen’s d.

## 3. Results

Thirty-two subjects were randomised and of these, 17 were allotted to MR-CBT and 15 to TAU (Figure 1). In the MR-CBT group two subjects were never treated, while in the TAU-group the corresponding figure was three. The treatment in the TAU group varied and included three cases of CBT, four used a psychodynamic approach and five used a general supportive intervention. The subdivision of the group by severity of depressive symptoms resulted in three groups: mild (SMFQ 8–13) n = 7 (26%), moderate (SMFQ 14–19) n = 13 (48%), and severe (SMFQ 20–26) n = 7 (26%), and there was no statistically significant difference in this distribution between the treatment groups. In the treatment sample there were 19 females, seven males and one of undetermined gender. There was no significant difference between the therapy groups in this regard.

The mean age was 14.2 years (SD 1.1) in the MR-CBT group and 15.2 years (SD 0.9) in the TAU group. The median number of MR-CBT sessions was 12, lower quartile 10 and upper quartile 17, and the median treatment period was 8 months, lower quartile 7 and upper quartile 11. With TAU, the median number of sessions was 20 times, lower quartile 15 and upper quartile 27, and the median treatment period was 8.5 months, lower quartile 5.5 and upper 11.

Fifteen subjects (56%) had a suicide event at the start of treatment, eight in the MR-CBT group and seven in the TAU group. In comparing the groups, the proportions were non-significant (*p* = 1.0). There was no subject with a suicidal event in the MR-CBT group after the end of treatment (*p* < 0.01). In the TAU group, three subjects had a suicidal event after the end of treatment (*p* = 0.2). The proportion of suicidal events after treatment was not significantly different between the two tretment groups (*p* = 0.07). By changing the cut-off for suicidal ideation to C-SSRS Item 2, nonspecific active suicidal thoughts, one subject in the MR-CBT group and five in the TAU group would be considered as having a suicidal event at the end of treatment.

Five subjects were administered a selective serotonin reuptake inhibitor, four in the TAU group, whereof one partially remitted, and one in the MR-CBT, who partially remitted.

With both MR-CBT and TAU there was a significant reduction in pre-post SMFQ scores, without significant difference between the treatment groups. The pre-post treatment scores for WHO-5 showed a significant increase after both MR-CBT and TAU, and there was no significant difference between the treatments (Table 1). Analysis with the last observation carried forward did not produce a statistically different result.

There were ten subjects (67%) with partial remission of depression in the group treated with MR-CBT and three (25%) in the TAU group. The difference between the treatments was close to significant (*p* = 0.05). The within group change in partial remission was statistically significant in the MR-CBT group (*p* < 0.01) but not in the TAU group (*p* = 0.2). When a lower cut-off (SMFQ score <6) was used for analysis, there were eight subjects with partial remission (*p* < 0.01) in the MR-CBT group and three in the TAU group (*p* = 0.2); the difference between the groups was not significant (*p* = 0.2).

The within sample effect size was 2.2 in the MR-CBT group and 1.3 in the TAU group (per protocol) and 2.2 and 1.1 respectively (intent to treat analysis). The difference in effect size comparing MR-CBT with TAU was 0.4 (per protocol) and 0.6 (intent to treat).

## 4. Discussion

### 4.1. Suicidality

An important finding of this study was the reduced suicidality after MR-CBT. It was noteworthy that this effect was not noticeable to the same extent in the TAU group. There was no significant difference between the groups at baseline that could explain this difference.

A specific feature of the MR-CBT treatment is identification of the suicidal state and its regulation. The protocol enables the processing of strongly negative emotions in a safe way. The mood map in the protocol often showed suicidality in a particularly intensive anxious state of mind, as well as a mix of fear, rage, pain and grief. This might be of importance, as for instance, aggressiveness has been proposed to be an important marker of suicidality both in adults and in adolescents [38,39,40], and also adolescents with emotion dysregulation had a chronic trajectory of suicidal ideation [41]. The focus on emotional and behavioural problem solving in the protocol of MR-CBT might be important as difficulties with problem solving has been identified as a part of suicidality in adolescents [42].

### 4.2. Depression

We found that the SMFQ scores decreased significantly with both treatments. It can be concluded that both treatment groups achieved clinically meaningful change, both close to the cut-off point. The within group change in partial remission was statistically significant in the MR-CBT group (*p* < 0.01) but not in the TAU group (*p* = 0.2). The low number of participants was probably the reason for the difference between the groups not reaching full statistical significance. However, the 67% partial remission did not equal the results reported by Wolpe [30] (p. 75) who found that: “Out of a total of 210 patients who have had these techniques applied to them, nearly 90 per cent have been either apparently cured or much improved, in contrast with a percentage not exceeding 60 in almost all other reported series.”

### 4.3. Wellbeing

WHO-5 scores after both MR-CBT and TAU treatment were close to normal after treatment, without any significant difference between the treatment groups, however, the small number of observations in the TAU group makes this result difficult to interpret.

### 4.4. Limitations

This study only managed to recruit half of the planned number of participants and the small number means low power to detect differences between the two treatments as well as risk of bias. There was no follow-up period. A general difficulty with studies on depression is the large response to a placebo condition, indicating—at least with mild and mild/moderate cases—a powerful tendency to return to the mean, i.e., there is a natural clinical pattern of declining severity [43]. Thus, the results need replication in larger studies with a long follow-up period and they cannot provide the basis for clinical recommendations.

### 4.5. Mechanism of Change

The National Institute of Mental Health (NIMH) has proposed a matrix of functional constructs for mental disorders to be used in research, Research Domains and Constructs (RDoC) [44,45]. This study relates to the RDoC framework by mainly focusing on two of the functional domains in the matrix, namely, the negative valence system and the positive valence system. The proposed mechanism of change, improved regulation of negative and positive affect, was supported by the theoretical and practical concepts of memory reconsolidation and counter conditioning.

The decreased score in the depression scale after MR-CBT, with an effect size of 2.2, might be explained by a changed in the ratio between positive and negative affect since the method specifically aims at increasing wellbeing capacity. This so-called positivity ratio has been shown to predict wellbeing in individuals [46,47]. In the words of RDoC, this indicates a new, better balance between the positive and negative valence systems. This result is in line with the findings in memory reconsolidation studies which show improved results after exposure treatment of fear memories combined with memory activation [26,27].

In the so-called third wave of CBT (the last 15 years), there has been an expansion of treatment approaches incorporating aspects of bodily and mental reactions and the tendency has been to focus on moderators of change in a transdiagnostic perspective [48]. MR-CBT can be seen as a part of this development, with its emphasis on a transdiagnostic approach, and on mood regulation, with a specific treatment mechanism.

### 4.6. Advantages

The advantages of this study include the natural clinical setting without exclusion of suicidal subjects, the application of a systematized treatment, and a proposed specified mechanism of change. Goldston et al. [49] showed that suicidal ideation in adolescence had two different trajectories when followed through into adulthood. About 75% normalised within several years, whereas about 25% of participants had a negative trajectory with an increase in adaptive difficulties over the years, which were associated with traumatic sexual events, anxiety and depression. The results of this study indicate the possibility that a brief focused intervention using MR-CBT can diminish suicide ideation and therefore, perhaps change the negative trajectory of severe depression in youth and help with secondary prevention.

## 5. Conclusions

There is a clear need for secondary prevention of suicide by developing efficient treatments for severely depressed and suicidal children at an early stage. In this study, mood regulation focused cognitive behavioural therapy (MR-CBT) did achieve a significant reduction in suicidal events in depressed adolescents in comparison to treatment as usual. MR-CBT focuses on changing the negative emotional content of the suicidal state in a treatment construed safe context. Although the study was small, the results presented indicate that memory reconsolidation-based treatments should be further explored in the endeavour to enhance the secondary prevention of suicidality.

## Figures and Tables

**Table 1:** Scores of SMFQ and WHO-5 before and after treatment in the MR-CBT-group and the TAU group (per protocol analysis). Mean (minimum-maximum).

	MR-CBT			TAU			
Effect variable	Baseline	End of treatment	*p* within sample *	Baseline	End of treatment	*p* within sample *	*p* comparing treatments ^#^
SMFQ	16.9 (11–23) (n = 15)	6.7 (1–17) (n = 15)	<0.01	16.4 (8–26) (n = 12)	8.8 (2–21) (n = 8)	<0.01	0.2
WHO-5	28(4–68) (n = 14)	63(24–92) (n = 14)	<0.01	32(4–60) (n = 7)	69(48–76) (n = 5)	<0.05	0.6

* Difference in score baseline/endpoint Wilcoxon matched pairs test. ^#^ Difference in score between MR-CBT and TAU at endpoint Mann-Whitney U-test.

## Supplementary Materials

The following therapy manual is available online at http://www.mdpi.com/1660-4601/15/5/921/s1, MR-CBT (Mood regulation focused cognitive behavioural therapy) the plus-minus-plus method.
