# Supplementary material for: Mood Regulation Focused CBT Based on Memory Reconsolidation, Reduced Suicidal Ideation and Depression in Youth in a Randomised Controlled Study
Source: Int J Environ Res Public Health. 2018 May 5;15(5):921. doi: 10.3390/ijerph15050921 (PMC5981960; doi:10.3390/ijerph15050921)
Supplement: Supplementary file 1 [file ijerph-15-00921-s001.pdf]

# MR-CBT
# (Mood regulation focused cognitive behavioral therapy)
# the
# plus-minus-plus method

Based on memory reconsolidation
and counter-conditioning

Therapy manual 2016

Göran Högberg PhD

## About the manual

MR-CBT builds on working directly with emotional regulation and in that work transforming memories so that the negative feelings and destructive impulses that are a part of the patient's difficulties are altered in a positive way. This transformation builds on memory reconsolidation by first activating positive emotions in the patient in order to achieve an affirmative mood in the treatment situation, then working on current and historical negative experiences so that they lead to less negative reactions and become less linked to destructive impulses. Each session is concluded with the creation of a positive emotional state. Finally, these interventions lead to new, more beneficial recollections that are consolidated in the long-term memory. This approach also has similarities with counter-conditioning therapy , psychotherapy by reciprocal inhibition, as descried my Joseph Wolpe 1958.

This manual presents a way of achieving these changes, if used with clinical discretion, that is, by adaptation to the treatment, to the patient and to the therapist. However, for the treatment to be said to follow the manual the main features of MR-CBT must be adhered to: as long as the basic principles are abided by, the techniques used can be other than those suggested in the manual.

In the following, PRM stands for Perception, Reaction and Motor impulse, a description of what happens within an individual when an emotional memory is active, also referred to as the PRM complex. Four-way processing is a way of describing how an emotional memory needing to have its negative charge altered is processed in four steps, four times: (1) focus on perception—the senses; (2) focus on bodily reactions—feelings and emotions; (3) allowing and acting out impulses—the inhibited actions; (4) recreating the event as it should have taken place.

*The language used*

You may perhaps start out with using the language in the manual, but will soon find the words that harmonize with your own voice. Above all, you will find yourself adapting your language to the person you have in front of you and the way in which you work together. Whatever words you use will be right as long as the main principles are followed, for that is what is most important.

## Preparatory work

*Informed and cooperative spirit*

You should stress the importance of explaining each moment of the therapy, in order that the client is well informed. The client should also be told in advance when you are introducing a new moment in the therapy and the client is explicitly asked if he/she would like to experience a new step. It is a question of safety and the client is to be trusted to know when to move forward and when to stay in the progress of the protocol.

*Counseling (psycho-education)*

When it is revealed that there are negative experiences in the patient's history I sometimes sketch a picture and talk around it. I may ask the child whether it knows of an animal that showed fear and to describe how it reacted, then go on to say that animals—like humans—have a number of reactions when faced with danger or have cause to feel fear. The first response is to freeze: hold your breath, remain still, perhaps feel some sensation in the throat and stomach. This first reaction is in order to find one's bearings and prepare for some form of action. Then follow the different action alternatives: hide, flee, fight, distract by appearing to be hurt, or ”playing dead” (dissociation, to mentally leave the body). Every reaction has a pattern, a motor program. This is general information. Specific questions follow: What reactions and impulses did the patient feel during the negative event(s)? Is there any correspondence between the patient's current disorder and these reactions to the negative event(s)?

After such an activation of negative memories, that is, even after only some brief talk about them, a delayed anxiety reaction may appear after a day or so. It is important to inform the patient about this phenomenon and to ask whether this has happened before and what, in that case, happens. One can then talk about how this delayed reaction can be understood and taken care of.

*The mood map*

At the first or one of the first sessions I encourage the patient to draw a mood map.

1. The mood map is a way of revealing in pictures the different emotions that have played out during a certain period. Generally I take the past week; but it can also be another period further back. As material I use A3 sheets and felt tip pens of different colors.
2. The instructions are open, some draw circles, some draw shapes, some make circle diagrams, some only write text and some use only one color. It really makes no difference, as long as the idea is understood. Sometimes patients decline to do the drawing themselves and then I ask whether I may draw while they tell me their story. This generally works as well.

3. First, the patient is asked to place him-/herself at the center of the paper, or as I used to say, that part of you that is always there, whatever your mood. This depiction is sometimes a circle, sometimes a name, sometimes a figure, sometimes a color.
4. The patient is then encouraged to position their different moods on the paper, larger or smaller in size, closer or further away from the center, to represent quantity and strength.
5. To complement the mood map I sometimes ask a few in-depth questions; perhaps to cover the “four big ones”: sadness, anger, fear, joy. It is good if these are included, but it must never be by demand, although an open question is never wrong, such as “did it ever happen during that time that you were really angry?” If I know that there is suicidality or brooding or some other symptom I ask where this is located, in what mood, and often get an amplification: ”black hole”, ”worst panic” or some other description with a corresponding figure for the symptom-producing state.
6. I also ask about the existence of any positive basic state, like simply feeling joy at being alive. If this is not a current state, but has existed at some previous time, the patient may for example place it far out in a corner, but it is important to highlight that even this mood exists or has existed.
7. When this basic structure is completed, the patient draws lines and arrows to show how the moods shift, and in what sequence, and how movement takes place from one mood to another. Here I do some investigation, initiate a conversation. An important question is how the patient manages to escape the most difficult mood states, for example, self-injury to the body, like cutting the arms to overcome some other pain. Another vital question is by what process a positive feeling is aborted and replaced with a negative one. A crucial third question is, what state of mind operates the complex of negative feelings? Sometimes brief texts complement the arrows concerning the shifts between emotional states.
8. The next step is to make a mood map for a future with the mood balanced; as it should be. This often takes little time and tends to be simplified, but very distinct. With or without drawing lines and arrows, the mood map of the future is often very clear.
9. The third stage is to place mood maps 1 and 2 in a row with a blank sheet in between. The question now asked is: what needs to be done to get from today’s mood map to the map of the future? Here the first response may be silence and uncertainty. I then say that only a few words or sentences will do or even just a question mark. The result most often is indeed a few words or sentences or sometimes just the question mark, but sometimes a new mood map is drawn.
10. In summary, the first mood map provides an external figuration of current emotions and their regulation. This in itself can have a calming effect, since moving from ”driver´s position”, when submerged in one’s feelings, to ”observer’s position”, when able to view

them from the outside, and is a way of achieving distance and gaining an overview. The mood map of the future gives the chance of formulating the possibility of another future in terms of mood regulation and well-being, while finally the third picture—the way forward—provides a treatment plan.

*Theory*

Depression in young people is about having difficulty with holding on to positive feelings and getting rid of negative ones. It is therefore important to understand that positive and negative feelings can co-exist. The problem then is mood regulation. Where depressed persons differ from non-depressed persons is lack of belief in a positive future; depressed persons find it more difficult to imagine that things can become better or that the future can in any way be good, since they more easily fall into thinking that everything is too late, already failed, too dismal. The mood map is a comprehensible way of portraying mood regulation and in principle always is appreciated. Patients feel able to communicate their inner experiences. It is also therapeutic in itself; the issue being not whether one is sick, but how to regulate one's moods, something that everyone has to deal with to some degree. The creation of a future picture generates the idea of possible change; gives hope. The formulation of what is needed to get there generates a readiness for action. By depicting them on paper the mood map gives the emotions a symbolic form and traces their interaction, which can help in controlling undesired mood swings.

**Affective brain training**

The core of the work is to practice, train the brain, to hold on to positive emotions and memories and to let go of negative affect more rapidly.

**The active work of change**

When the treatment is under way with regular sessions these are often commenced with a Plus/Minus map, with one column detailing recent positive events and the other negative ones. The theory involved is that present day joy and adversity are connected to deep strata of positive and negative emotions and life-experiences and that these are affected also by working with present-day  plus and minus affect. This is also a way of connecting to what the young person finds important at the moment.

*Approach*

Be directive but give open instructions: ”You can…; one can…; it can happen that…, etc.”. Your role is to participate when something transpires, much in the way of a midwife or a

gardener. A motto might be: trust the process. Your task is to initiate, then to back off and let events develop, but keeping a close watch on what is taking place, ready to offer support and to assist with transitions to new phases. Imagine that you are actively in your consciousness switching between the patient's breathing and inner experiences and your own breathing and inner space.

*Support*

The most important support is to help and monitor quiet relaxed breathing during the session. Such support can have a calming effect that increases trust in the treatment situation—a way of introducing information about external support and surrounding safety as a counterbalance to the inner anxiety which old, negative memories can induce. This is about training in the tranquility system in order to offset the activity-alertness-anxiety system.
Another support is to as soon it is possible have a chosen person to be present with the client, be it a parent, other adult. With adults a spouse or friend can be of great help as support, also the shared experience creates positive bonds.

*Creating a positive feeling*

The basis for the continued work is creation of positive feeling. This feeling must be now, in the room, in the patient's body. To obtain a positive feeling in the patient demands active work with different techniques

1. Breathing and relaxation exercises. I usually ask patients whether they have had any experience of this. Some have practiced relaxation in school, some in music or gymnastics classes, some not at all. Then I talk about emotions, the control of emotions and how breathing relates to this. I say that everyone can find their own ways; mine are only suggestions, but they can also be used at home. I also say that it is a good idea to practice breathing and relaxation when one is calm and feeling well, so that they are already in place as a handy resource when one is not in such good shape, when it is easy to lose sight of such knowledge. Breathing is also a way of communicating states of mind—moods—between people.
2. The next thing I do is carry out three exercises. You can of course here use your own experience and apply whatever you think may work. The scope for variation is wide; what is important is that whatever you try fits the specific patient.
3. **Conscious breathing**. I say: "Sit comfortably and be conscious of your own breathing. You may choose whether you want to keep your eyes open or closed. Concentrate on your breathing. Think: Here I am, at this moment, and this is what it feels like to be sitting here and simply breathing. When other thoughts come, whether in the form of pictures or voices, take note of them, but then let them float away next time you exhale. Continue to

direct your mind to the thought that this is how it feels, at this moment, to me precisely, when I breathe." After a while you might add: "You may now be feeling different sensations in your body—lightness, heaviness, tension, relaxation, warmth, chills, or something else. Take note of your bodily reactions, then let them pass out with each outward breath. Then go back to the thought that this is how it feels right now when I breathe." After a while the patient is generally left to do this on her own, to concentrate her thoughts on her breathing and then observe and expel other thoughts and bodily sensations as they arise. This phase tends to take the longest time, since it gives a chance of discovering what inner images, thoughts and bodily sensations are making themselves felt and whether it is possible to influence them. If it is difficult to subdue worrisome images, thoughts and sensations (which sometimes even increase to start with), the breathing exercise is repeated and often the problem is solved after three rounds or so.

4. **Safety measures**: if the inner images and bodily sensations are so strong and terrifying that they cannot be controlled with conscious breathing then another program is required that is focused on movement, breathing and mental reinforcement. This is developed in item 9 of this section.
5. **Progressive relaxation** in a much shortened version can be used. Here it is important to include the facial muscles as these are connected to both expression and experiencing of feelings.
6. **Balance breathing** is a modified variant of Thich Nhat Hanh's meditative breathing: count on the inhalation; count on the exhalation; find a rhythm. This can be done while walking up and down the room or outdoors; the walker focusing on the contact between the foot and the ground while assuming a slight friendly smile. Balance breathing has the advantage of being easy to learn and to produce when needed for mood regulation.
7. From there I sometimes proceed directly to **artistic breathing** and say: Imagine something artistic or from the natural world, a melody or other sound, or something to do with movement—maybe a line from a poem, a musical theme or something other with movement in it—but with a *rhythm* to support your calm, safe breathing.
8. ***Note to yourself***. Often it is beneficial to concentrate on your own breathing at this point and to synchronize it with the patient's.
9. The work is with regulation and control of inner and external experiences. If the inner are too strongly disturbing and hard to control, it is important to help the patients in their effort to exert control by strengthening external sensory input. A first endeavor may be to get the patient to stand up and be aware of contact with the floor, the pull of gravity, the ambience around, sounds, visual impressions. It is often good to walk about, either within the room or outdoors, and while walking to practice breathing that is in rhythm with

the steps taken according to the meditative walk developed by Thich Nhat Hanh. It is possible to develop this walk with sensory activation so that it becomes a sensory walk where the senses are activated, one at a time; for example, that for a while we only notice what we hear, then what we see, and finally what we experience in our bodies. There are other ways, such as listening to music, painting or drawing, or working with the hands, anything with a pronounced presence in external sensory impressions.

*Breathing*

At every stage it is important to work with deep, calm breathing; there are often trapped feelings in the final expiration of deep breathing. A deeper and more open breathing in the upper part of the chest is associated with feelings of greater calm and of inner freedom.

*The safe place exercise*

10. Anchorage in a safe place. This is a self-help technique spontaneously used by many people, but which also has been formalized in different therapy contexts. The aim is to achieve mood change by removing oneself into a positive imaginary space. First ask the patient if s/he can imagine a place where s/he feels calm and safe, a place that exists now or that has existed previously, or that s/he simply can visualize. Sometimes it is easier to think of a person or an activity, and sometimes when it is really difficult this may be the treatment room itself, or the way to and from the treatment sessions. Sometimes nothi ng will come up or there may be several choices, in which case give open instructions and see what transpires.
11. Now comes the inner activation: ”Imagine yourself in a  film or a daydream, or that you’re a character in a good book. Open your inner senses, see what this place looks like—the colors, the furnishings, the surroundings—here you are calm and feel safe and in control. Hear the sounds, feel the rhythm. Notice: is there any aroma? Notice the feelings in your body; the feeling in your stomach of calm, safety and control.” Here a question may be inserted about any movement, action or rhythm connected to the feeling of calm, safety and control.
12. This is followed by instructions to breathe deeply and calmly. At times safety can be given by the parent holding the hand of patient  What is aimed at here is a safeguarding sensory stimulation that confers calm from without and a feeling of contact with the world around and with another person.
13. Finally, to consolidate the feeling created of a safe inner place: ”Take deep, calm breaths and let the feeling of calm and control fill your whole body,”  this is a practice of retaining positive emotion.

14. The next step is to get the patient to use this capacity: ”You now own a resource, the ability to think of something that makes you feel safe and in control. Now imagine that this can be stored away; put in a place where you know that you have it, to take out when you need it and where you can keep it when you don’t need it any more. It will be your secret hiding place, maybe somewhere out in nature, in a pretty little box or in a part of your body. Imagine putting that feeling away and then bringing it back. Train yourself to let it come, like watching a film.”
15. ***Comment***: How much do you have to know? Often I get no more than a general outline of the safe place. I say that you can describe it to me if you want to or not if you don’t. Sometimes the patient chooses not to tell. It doesn’t matter either way, in fact, not telling saves time. The important thing is getting the inner imagination, the sensory system and the positive autonomous reactions started.
16. Sometimes the attempt to create a positive feeling fails. The thing to do then is to begin again and work some more on this. Talk about what it is that feels uncertain and insecure, perhaps bring a trusted person into the room, perhaps stand up to strengthen the feeling of balance and other sensory impressions, perhaps go for a walk, or use an aerobic treadmill or trampoline, or some other form of physical activation. Important is to let the process take its time. Without having first built up a positive feeling it is impossible to go on and create a positive association that can be stored and attached to the negative memories.
17. ***Your own position***. Enter your own parallel picture based on what you glean about the patient. Share your attention between following the manual, observing the patient, observing your own inner reactions, and following the process. Give open instructions, such as “it can happen/it sometimes happens that…”, ”one can also…”. From time to time observe what happens with the patient’s upper chest.

*Creating a positive self-image*

Go on to a positive event that has recently taken place. This may have emerged from the plus/minus chart or else now put the question. Get the patient to visualize this event,: ”See yourself in that situation, when you make good, when things work out and you are happy. *Be* yourself in that situation and re-experience that truly happy feeling” (that is, a change from ”observer´s position” to ”driver´s position”). Now add the thought: ’What does this say about me?’ and experience this as a signboard or a voice.” Here the patient sometimes chooses both. ”Now view the situation once again and add words that express a positive determination of self esteem.” Then ask what is happening in the body, supported with calm breathing. Expressions of joy may appear here—giggling, a sense of pride, sometimes a swelling of the upper chest.

*Negative feelings*

It is now time to ask whether it is possible to look at the negatively charged emotional memories.

1. I ask, ”Shall we now look at those negative feelings and experiences?” If I have a plus/minus chart I can go into the negatives. Sometimes the patient says no and then we continue the session with building up positive feelings, e.g., a person liked or something good the patient would like to see happen in the future. Often, however, the patient is now ready to face up to the negative feelings. [Here one must oneself take a deep breath and prepare to be involved in other moods.] The technique is simple if following the PRM complex principle. First, “See the event as you remember it, what did your senses note? What did you see? What did you hear?” After going through the sensory experiences, say, ”Imagine that you are a film director or a storyteller and remake the film, or retell the story. Enter the event yourself and feel what reactions come, what is happening in your body—the tension, the pain, the nausea, the breathing difficulties, the increased heart rate, etc. what feelings do you recognize? Play the film from the beginning.”
2. This is followed by the action stage. ”You are the director/creator of the story, now replay the scene. You are yourself part of what is taking place and this time you allow all the inhibited movements, impulses and actions to play out completely. Take the film from the beginning and let the movements and actions come.” This can often be liberating, but sometimes elicits the reaction to hide, to tense up, become immobile. One must then encourage the held back impulses and strengthen the muscular forms of expression.
3. Following this it can be fun to repeat the movements in the form of an animal; say, a roaring lion, or a galloping horse. Ask the patient to suggest an animal to express the movement: ”Imagine that you’re this (horse). Become the (horse). Enter the feeling of racing along…”
4. It is now time for the fourth repetition; this time a positive imaging. ”Conjure up in your mind how it should have been. Imagine that someone else enters the frame, or that you or someone else does something different from before; as it should have been.” This is the stuff of daydreams and literature—the creation of new, alternative scenarios—and is about both training the imagination and creating positive memory traces.
5. These are the basic steps. Based on the first memory image and the reactions provoked by four-way processing there may sometimes be reason to enquire whether there is a connection to some earlier first event that introduced the same feelings. Sometimes this emerges directly. Sometimes it can be elicited by embarking on a journey: ”Imagine that you are flying with this feeling, like a bird, imagine yourself gliding over your life. Now see if you can remember when you’ve had this feeling before. When you come up

against something, maybe you can go down and investigate what that was about? Then go on looking. Whenever you find something: stop; consider." Then we do a four-way processing of the PRM complex that is activated.

6. When an associative chain to earlier memories has been established, these previous templates for the present disruptive emotional reactions need to be dealt with.

*Concluding the session*

Towards the end of the session it should begin to feel as if an atmosphere of calm and closure has been established.

1. To conclude the session, go forward in time. Ask the patient to create a new inner movie or affirmation that everything will go well until we meet again. Replay the movie/repeat the affirmation a few times. Possibly reinforce with sensory stimulation.
2. The patient now returns to the safe place, preferably by own effort, but otherwise of course with gentle encouragement.
3. Allow a few minutes rest in this place with no instructions whatsoever. This is a way of becoming 'grounded in oneself', and gives a chance to digest the different impressions newly experienced and to prepare for whatever is waiting after the session.
4. Ending the session: Evaluation; How did this work for you?  Thoughts? Comments? Questions? If there was an accompanying person along ask for his/her experience during the session.

**Some variations to use when the problem is formulated differently than a specific disturbing memory.**

*Falling asleep, sleep, rest*

Depressed and anxious patients often have difficulty falling asleep. Relaxation can bring on anxiety, and they find their heads filled with irritating ruminations. They may be afraid of falling into nightmares, memories of negative events may surface, or they may simply find it difficult to fall asleep without quite knowing why. A 24-hour graph as a sine curve is useful for eliciting a microanamnesis of the sleep-wake cycle. A lack of sleep several nights in a row can be devastating for daytime functionality, since concentration ability, learning ability and mood regulation are all then strongly influenced in a negative direction. *Insomnia*, therefore, or any tendency in that direction is *the first problem to be tackled*

1. Identify the source of disturbance. The basic problem of course is that body and head are out of synchronization. Fatigue signals are not reaching their goal; instead anxiety

signals take the upper hand and increase wakefulness. The task here then is to decrease the anxiety signals and increase the fatigue ones.

2. Does the patient apply own tricks to try and solve the problem? What works? What doesn't?
3. Concerning 'sleep hygiene' I have found that the most important remedial measure is as far as possible to remove all access to visual input, such as TV, PC, cell phone, lights.
4. Next to contend with are the inner, mental distress signals. Is it about fear of nightmares; anxiety or pain in the body; bad memories resurfacing; worry about tomorrow; grief over some loss?
5. Whatever the theme, it can be processed with relaxation and breathing exercises, and by taking refuge in the safe place, using the PRM complex methods with four-way processing. Concentrating on the falling asleep situation itself is often productive for retrieving the appropriate theme.
6. Having worked on the hindrances, sleep training can then take place through breathing and relaxation exercises, then locating, feeling and allowing the fatigue signals to spread throughout and be accepted by the body. ”Feel the tiredness in your body. Think that activity requires rest, that in rest you gain energy and can charge yourself for new activity. Feel in every part of your body how willing it is to rest, how every cell opens itself to rest so as to gain new energy.” It may sometimes feel appropriate to insert a kind of 'heart meditation' with focus on rest. ”Feel your pulse. Your lungs are taking in oxygen that is stored in your blood. Your heart is transporting that oxygen to every part of your body, to every cell in your body, which now, as you rest, is being charged with new energy.”
7. Now take a longer pause—five minutes or more—while you slowly and calmly repeat: ”Feel your fatigue, accept the need to rest, if you fall asleep that's fine, I'll wake you when it's time.”.
8. A further exercise for sleep training is the following: Let the patient perform horizontal eye movements with closed eyes and calm breathing to muscle exhaustion, followed by a short rest, then a new bout of eye movements to exhaustion.  This may, but need not, be coupled to counting sheep (☺).

*Dreams*

It is important to be able to work with both normal dreams and nightmares. Dreams are stories that mirror either earlier or current events, or concurrent experiences during the night, along with different sorts of learning. Nightmares can be very frightening and correspond in the feelings conveyed to anxiety attacks during the day.  Begin according to the manual, conjure up the dream and work on it with four-way processing. It is sometimes of value to

investigate possible connections to earlier episodic memories, which sometimes emerge spontaneously and sometimes as the result of questioning, and then apply four-way processing.

1. Here a few words about what dreams may contain. No complete description is necessary, except the theme and the feelings attached. Ask also whether the patient has worked with dreams previously and whether s/he holds any theories about the importance of dreams.
2. ”Imagine the dream. See yourself as the movie director who can form and develop different versions of the same story.
3. After each finished version, create a movie or series of images of the dream, see if you can go in and become a part of it, or if you’d rather, view it from the outside. Play the first version where you stress your sensory perceptions. What is it like? What are the sounds? The movements, etc.?
4. Now make a new version. This time with your own bodily reactions. What do you feel? How is this evident in your body?
5. Make a new version. This time make a note of your movement impulses in the dream. Live them out, exaggerate, you can do whatever you like!
6. Make a last version. This time you dream the dream as you wish it had been/as you want it to be. It can be a new version of the old dream or something completely different.

*About disruptive thoughts*

Often patients have problems with finding peace at night because their head is brimming with disruptive thoughts and the body is unable to relax. Sometimes these thoughts are clearly defined—a recurrent memory, a flashback from a negative experience. But other times it is the sheer tangle of thoughts, the way they disturb, the speed of their shifting that is the problem. It is then important to first get a grasp on the character of these thoughts and the manner in which they cause the patient concern. If it is the situation of tumultuous thoughts itself that is the difficulty, and that repeats itself and becomes entrenched, then this is what must be the focus of the four-way processing. If the traumatic memory comes from the situation of disturbance itself, I may say something of the following: ”imagine a situation where you are troubled by your thoughts, go through them, maybe frame by frame, or perhaps let the pictures flow, like a movie, and note what you perceive with your senses, what you see and hear. Good, now run the film again, and this time make a note of the feelings provoked, how you react and where in the body it is located. Good. Now repeat the process again, but this time release the impulses that are trapped inside. What does your body want to do? Imagine that you allow yourself to do these things. Let the film show how

you act out your impulses. Good. Now go back to the situation where you are disturbed by your thoughts, but recreate it in the way that you wish it to be."

In this way, the situation itself is treated and how the thoughts distress, rather than working on any specific theme. However, out of this process specific memories may arise that can then be treated separately.

*Relationships: alternative with inner processing*

In working on mood regulation, relationships are always important. Both positive, with longing and comfort; and in loss, with longing, seeking, grief, pain and vulnerability; as well as experience of violence and abuse, with fear, avoidance and aggression as defense, and dreams of revenge.

There may be strong feelings attached to important persons. The PRM complex is then activated and can be processed in a fluid manner. When an important relationship comes up, the process is always the same: to **go into** the experience. I say things like, "Choose a place where you can come face to face with this person/these persons. If you can't find a place, never mind, just visualize the person. Look that person in the eye; make a note of what you perceive. Talk to the person; say what you need to say—the underlying things beyond the usual conventional words. Practice saying 'yes' and 'no', what you want, what you don't want. Try engaging the person in a dialogue. Imagine that you hear what the other person is saying and understand what s/he means. [Sometimes it works to enter a dialogue, sometimes not.] Now when you become active in this relationship, what reactions do you observe in your body, what are your emotions? And what do you spontaneously feel like doing with this relationship as you experience it just at this moment? Let this now happen in your imagination. You can tell me if you like or keep it private. Now, if this feels like a possibility, imagine your relationship with this person if life was what it ought to be. How would you like your relationship with this person to shape up in the future? Make a wish and imagine that wish fulfilled.

*Grief*

Pathological grief tends to be viewed in terms of a dichotomy: either the rigid shutdown where it cannot be spoken of, is denied and kept at bay; or the overflowing grief that with no effort at control fills all of life. An important loss does not disappear. Rather, it becomes integrated in the personality. The goal is to create control over this process and to find approaches that allow an inner, living dialogue with the deceased, or the lost time, in a way that offers support to maintain the relationship, but in a controlled dialogue. The technique is the same as in work with living relationships, with the addendum of the attempt to manage the terms of the inner dialogue so that ongoing life is sustained.

*Early decisions about facing life*

With repeated strong feelings of betrayal, insecurity, violence and disappointment in early childhood, lasting features may be created in the personality. This may appear in MR-CBT as thoughts and decisions related to pictures of early negative events. Such decisions may be: ”there’s no point in expressing own wishes;” ”I can never trust another person;” ”it’s dangerous to show feelings;” ”I can protect my mom by being unruly;” ”by being submissive I can keep my mom from changing;” ”I have to wait my turn;” ”it’s all my fault;” ”everything would be fine if only I were nicer”, etc. in any number of variations. Especially relevant is this type of determining and sensitive decision at preschool age, often surprisingly early, if the child’s parents suffer from substance abuse or mental problems, where children tend to take on a huge responsibility, far beyond their years, for protecting parents and siblings; a sort of contract with life about what attitude will work best.

Here it sometimes is possible during the current inner processing to let the patient at present age meet the traumatized child and together discuss whether it is possible to renegotiate the contract.

*Taking care of the inner child*

This is a technique that now is common in many forms of therapy. It is carried out as above, although not about the contract, but rather, ”What can you do? How can you explain to and take care of that part of you that is trapped in that memory of early negative events?”. This can be included as an element in the four-way processing called ”as it should have been—an imagining that opens up for other possible scenarios then, and thereby for the future.

*Difficult choices*

Ambivalence and decision anxiety are normal. This can even be true of negative symptomatic behavior: ”Shall I have another beer or not, smoke another cigarette or not, go on a diet or not, stuff myself and throw up or not, cut myself on my arms or not?”

1. One way of beginning may be to investigate the PRM complex at the moment of deliberation itself and work on it with four-way processing.
2. Often the chance will come up to investigate each moment of deliberation independently: ”Enter the movie you’ve created and see what happens if you do A, then make a new version and see what happens if you do B.”
3. It may be fruitful to let the alternative choices, personified, speak or interact with each other, on the same stage, at the same time.

**Alternative external processing: dialogue techniques**

In the previous part of the manual we have dealt with imaging and body-experiences. In the following part we look at some examples how the principle of memory re-consolidation work can be used in inter-personal communication.

Dialogue techniques naturally have their own place in the theater, but can be used in therapy using direct address and dialogue methodology. Dialogue develops reciprocal feelings and interpersonal interaction, and stimulates mutual intuition and understanding. Here follows a simple technique, working with the relation of an important person who is not present in the room: ”Imagine that the other person is sitting on a chair (or if a group, several chairs).” Other objects or symbols such as dolls or animals can function just as well, but are better used with smaller children. The chair is suitable for teenagers, although it also works to simply imagine the person as projected on the wall. Then begin the ”speak to” process. Let it develop and intensify in its own time, with only gentle support, if necessary. Try introducing the main idea of MR-CBT, that is, to first find the positive in the other, only then to go on to the negative. If the feelings that arise are strongly negative there may be reason to first speak to someone who gives a feeling of security. Just as otherwise in MR-CBT, it is necessary to first create an atmosphere of safety and calm before negative emotions are activated, and to end with conjuring up a picture of how the patient would like the relationship to shape up in the future. It can happen that the patient's address is conducted silently within herself, but looking and directing the words towards the chair.

**The patient must address the other directly.** This is the basic technique. It can be developed with support techniques such as pairing with the patient and giving gentle assistance to say what needs to be said, or you may assume the opposite role and improvise a dialogue, which can be very productive and surprising to the patient how much you capture correctly. When using these aid techniques one must naturally get feedback from the patient; how close was your interpretation of the other?

A positive closure of such a session is to let the patient experience the dialogue from ’the outside’. That is, the patient is the audience as you assume and act out both roles. The patient is then asked to comment on what s/he has seen.

*Relations, alternative with external processing*

This is a way of deepening the dialogue in child-parent, child-child or adult-adult conversations, when both parties are present in the room. Let the two persons sit on chairs placed opposite to each other. Diagonally behind each person place a 'helper' chair. Now encourage the persons to speak to each other, say; “You are now in a dialogue invitation position. Stay in control,” perhaps suggest a theme for the conversation. When you feel that it might be useful, go to a 'helper' chair, say something like: “When I sit here I am trying to

guess what you are feeling and what you want to say. I may be quite wrong, so after a while I will ask you if what I am saying feels right, and you can also interrupt me whenever you like and tell me if it doesn't feel right." Try to synchronize your breathing with the patient's and to catch the subtexts beneath the surface that still haven't found expression. It is important to switch between 'helper' chairs and always to investigate afterwards how well what you have formulated is in agreement with the patient's own perceptions.

Themes may balloon back and forth, but I lead the interchange so that they first try to say good things to each other, then follows space to express dissatisfaction, and finally to explain to each other how they wish the positive future of their relationship to unfold. Important is always to end with a normal conversation. Any embarrassment caused by the foregoing exercises tends to disappear when you are the one taking the risk of exposing yourself.

In the dialogues, it is of importance to follow the principle of memory reconsolidation, ie. first trying to dwell on shared positive moments, then there could be room for conflict resolution and finally it is possible to go together in a positive future projection, creating a positive memory trace.

**Family counseling with the stepfamily system where several families are involved. Separation and attachment based family therapy (SAFT)**

This is about supporting the child/teenager in a situation where several families are involved—the stepfamily problem complex. Such sessions are required when the stress arising in these constellations is judged to be harmful to the patient.

1. First talk alone with the patient. What is s/he experiencing? What does s/he want? What does s/he wish to avoid? Next, meet all those involved together. Provide a framework, explain that the aim is to help them to be more supportive of each other and so reduce the level of strain. Explain the structure and progression of the process they are about to experience. Take ample time, at least two hours or even three with a break in the middle.
2. In each constellation speak first about what is good, positive memories, signs of care and affection, memories from before birth, of early childhood, positive future trajectories; there should be an immersion into a bath of positive mutual feedback. Then allow the difficulties and irritations to come up say "it is not possible to change another person but can have your right to express what change in the other person would give you joy and meaning", Focus also on issues of separation and attachment, coming and leaving, entry, exit, change, planning or the lack thereof. This focuses the thoughts on the family

as a living constellation in a process of development through mutual collaboration. Then close by sharing mutual hopes and plans for positive future relationship.

3. Let the children meet with each grown-up, together or separately, to talk through what is good and what not so good, according to the protocol in a flexible way. In the case of younger children it may be easier to work with drawings, however.
4. Let the adults talk separately with each other, perhaps first only parent pairs with each other, later maybe all the adults together.
5. Let the children meet together without the parents,  just the siblings with each other, focus on their care and responsibility for each other and that they can have a good relation for their full lifes, spin on positive future fantasies.
   Finally collect everyone for a summing up, discuss the themes that have emerged, see if the group can arrive at some agreement. Sum up; use a flipboard or a whiteboard. Try to arrive at some decisions. Evaluate.
6. Make an evaluation of the session(s).

**SAFT with intact family**

How to do SAFT with a family that is still intact? You may choose to work with everyone in the same room at the same time or divide the family up in smaller components. Then follow the same protocol.

**Other modalities**

The plus-minus-plus method presented in this manual can also be used with other modalities such as talking therapy, art-therapy, movement therapy, drama therapy, music therapy and counseling. This manual can be tailored according to the tradition of the therapist and the needs of the client.

***Afterword***

Memory reconsolidation based therapy is about relearning and changing affect related to memories. This reduces the weight of the patient's experiences and heaviness of spirit, and actions become less steered by former negative experiences. Fundamental to the proceedings presented in this manual is to develop and maintain calm, rhythmic breathing. We can say that the treatment is borne up by the breathing—a breath-borne experience alteration process. By training well-being during the sessions, mood regulation is also practiced, leading to better control of negative feelings. According to PRM complex idea it is important to stimulate perception, whereof the orientation towards the different sensory systems: further reaction, the autonomous system, and motor reaction—the impulse to act—which explains the questions concerning movement.  The future journey—the exercise

simulating new successes to nullify past failures—is important for being able to create better visions of the future. Many clients see this form of therapy as a form of brain training, affective brain training to keep positive emotion and let go of negative. This can also be seen as a training of emotional creativity.
